# Fluids or vasopressors for the initial resuscitation of septic shock

**DOI:** 10.3389/fmed.2022.1069782

**Published:** 2022-11-24

**Authors:** Stephen Macdonald, Sandra L. Peake, Alasdair R. Corfield, Anthony Delaney

**Affiliations:** ^1^Medical School, University of Western Australia, Perth, WA, Australia; ^2^Department of Emergency Medicine, Royal Perth Hospital, Perth, WA, Australia; ^3^Centre for Clinical Research in Emergency Medicine, Harry Perkins Institute of Medical Research, Perth, WA, Australia; ^4^Faculty of Health and Medical Sciences, School of Medicine, University of Adelaide, Adelaide, SA, Australia; ^5^Department of Critical Care Research, Faculty of Medicine, Nursing and Health Sciences, Monash University, Melbourne, VIC, Australia; ^6^Department of Intensive Care Medicine, The Queen Elizabeth Hospital, Adelaide, SA, Australia; ^7^Consultant Emergency Medicine, Royal Alexandra Hospital, NHS Greater Glasgow and Clyde, Glasgow, United Kingdom; ^8^School of Medicine, Dentistry and Nursing, University of Glasgow, Glasgow, United Kingdom; ^9^Malcolm Fisher Department of Intensive Care Medicine, Royal North Shore Hospital, Sydney, NSW, Australia; ^10^Division of Critical Care, The George Institute for Global Health, University of New South Wales, Sydney, NSW, Australia; ^11^Faculty of Medicine, Northern Clinical School, University of Sydney, Sydney, NSW, Australia; ^12^Department of Epidemiology and Preventative Medicine, Australian and New Zealand Intensive Care Research Centre, Monash University, Melbourne, VIC, Australia

**Keywords:** sepsis, shock, fluids, vasopressors, hemodynamic resuscitation

## Abstract

Intravenous fluid resuscitation is recommended first-line treatment for sepsis-associated hypotension and/or hypoperfusion. The rationale is to restore circulating volume and optimize cardiac output in the setting of shock. Nonetheless, there is limited high-level evidence to support this practice. Over the past decade emerging evidence of harm associated with large volume fluid resuscitation among patients with septic shock has led to calls for a more conservative approach. Specifically, clinical trials undertaken in Africa have found harm associated with initial fluid resuscitation in the setting of infection and hypoperfusion. While translating these findings to practice in other settings is problematic, there has been a re-appraisal of current practice with some recommending earlier use of vasopressors rather than repeated fluid boluses as an alternative to restore perfusion in septic shock. There is consequently uncertainty and variation in practice. The question of fluids or vasopressors for initial resuscitation in septic shock is the subject of international multicentre clinical trials.

## Introduction

It is important to appreciate that many of the IV fluids in clinical use today were introduced into practice at a time when clinical trials were not standard and, thus, not subjected to the same rigorous evaluation as current drug therapies. It was not until the twenty-first century that a systematic evaluation of IV fluids in clinical practice began. Landmark clinical trials have investigated the comparative effectiveness and safety of albumin (1, 2) and synthetic colloid fluids (3). More recently attention has turned to investigating the safety of “balanced” crystalloid solutions relative to 0.9% saline (4–6). Another aspect of the use of IV fluids which has recently come under investigation is the question of dose, both volume and rate of administration, particularly in the setting of sepsis and septic shock. Current international guidelines recommend at least 30 mL/kg of fluid within 3 h as first-line therapy for sepsis-induced hypoperfusion or septic shock (7). Following “adequate” fluid resuscitation, vasopressors, preferably noradrenaline, are recommended to correct persistent hypotension/hypoperfusion with initial infusion *via* the peripheral route to prevent delays in vasopressor administration. Nonetheless, the evidence underpinning guidelines for early hemodynamic resuscitation is of low quality and the recommendations are graded as weak, leading to clinical uncertainty. The question of fluid dose and the relationship with early vasopressor administration as initial resuscitation in patients with sepsis and septic shock is the focus of this review.

## Physiological rationale for fluid resuscitation

Sepsis is a clinical syndrome characterized by life-threatening organ dysfunction caused by a dysregulated host response to infection (8). Septic shock is a subset of sepsis where the underlying cellular and metabolic abnormalities are profound enough to substantially increase mortality. According to the Sepsis 3 definition, septic shock is identified clinically as sepsis with persistent hypotension requiring vasopressors to maintain a mean arterial blood pressure of ≥ 65 mm Hg and a serum lactate level > 2 mmol/L despite adequate volume resuscitation (8).

### Pathogenesis of septic shock

The pathogenesis of septic shock is complex. The host innate immune response identifies molecular signals from invading pathogenic organisms and tissue injury leads to an initial pro-inflammatory response involving activation of leucocytes, complement and the coagulation pathway (9). At the same time there is a compensatory anti-inflammatory response involving neuroendocrine regulation *via* the hypothalamic-pituitary-adrenal axis, inhibition of pro-inflammatory gene transcription, expansion of regulatory and suppressor T cell lines and apoptosis of pro-inflammatory B and T cells. The relative balance of these elements is governed by a range of host and pathogen factors such as age, comorbid illness, genetic susceptibility, microbial load and virulence. It is the dysregulation of these immune responses which lead to the clinical consequences of organ failure and shock.

### The vascular endothelium and endothelial glycocalyx

The vascular endothelium plays an important role as an initiator of the sepsis response at the site of local pathogen invasion or tissue injury and as a propagator of the inflammatory response (10). Endothelial cells express Toll-like receptors (TLRs) which bind circulating Pathogen-Associated Molecular Patterns (PAMPs) and Damage-Associated Molecular Patterns (DAMPs). This initiates intracellular signaling and the transcription of inflammatory mediators. Activated endothelial cells reprogram into a pro-inflammatory and secretory phenotype, releasing tissue factor, von Willebrand factor, and inflammatory cytokines such as interleukin-6 into the circulation. The endothelial glycocalyx is a thin (0.2–5μm), negatively charged, mesh-like layer of proteoglycan molecules anchored to the luminal surface of endothelial cells to which are attached sulfated glycosaminoglycans such as heparan and chondroitin (11). The polysaccharide hyaluronan is embedded within the glycocalyx *via* attachment to the CD44 receptor on the endothelial cell surface.

In health, the endothelial glycocalyx maintains a barrier between the circulation and the vascular endothelium, while its physical and electrical properties are important in maintaining the oncotic gradient, preventing large molecules such as albumin from crossing into the sub-glycocalyx space. Shedding of the glycocalyx, which occurs during inflammation or ischemia, leads to loss of this barrier function. Activated endothelial cells express intercellular and vascular cell adhesion molecules, and selectin molecules. Collectively, these bind to circulating leucocytes and facilitate the conformational changes to allow diapedesis of leucocytes and extravasation of fluid and molecules from the circulation into the tissues. Finally, the procoagulant state of activated endothelial cells leads to recruitment of platelets and activation of coagulation which can lead to microvascular thrombosis and injury (12).

### Cellular and metabolic changes in sepsis

In contrast to other shock states such as acute hemorrhage or low-cardiac output states, tissue hypoperfusion is usually not the primary mechanism for sepsis-associated organ dysfunction. Tissue hypoxia is less common and widespread tissue necrosis is not a typical post-mortem finding among patients dying of sepsis (13). Rather than impaired oxygen delivery, the metabolic changes occurring during the critical illness stress response alter mitochondrial function, leading to reduced oxygen utilization (14). Elevated lactate levels, which can predict increased sepsis mortality (15), may be due to mitochondrial dysfunction or related to catecholamine-induced hepatic production rather than anaerobic production due to tissue hypoxia (16). Some authorities have postulated that sepsis-induced organ dysfunction may be an adaptive, endocrine-mediated response to overwhelming systemic inflammation (17).

### Hemodynamic changes in septic shock

The classic sepsis phenotype is distributive shock with peripheral vasoplegia and preserved or increased cardiac output. In practice, there may be a combination of peripheral vasodilatation, hypovolemia due to reduced fluid intake and extravasation and depressed myocardial function secondary to systemic inflammation and acidosis (18). Peripheral vasodilatation occurs as a consequence of increased nitric oxide production, reduced vasopressin production and downregulation of vasoconstrictive receptors for angiotensin, catecholamines and vasopressin on vascular smooth muscle (19). These changes can occur as a severe manifestation of the generalized systemic inflammatory response to localized infection. Additionally, exotoxins produced by pathogenic gram positive bacteria act as “superantigens” cause widespread activation of the inflammatory cascade e.g., “toxic shock syndrome” (20). Lipopolysaccharide endotoxin released by lysis of gram-negative bacteria act as a PAMP, binding to TLR-4 on immune and endothelial cells and triggering intracellular signal transduction and activation of the pro-inflammatory cascade (21). Finally, alterations to the function of the hypothalamic-pituitary-adrenal axis and tissue resistance to glucocorticoids in sepsis can result in absolute or relative hypo-adrenalism (22). Administration of glucocorticoids results in faster resolution of shock in ventilated intensive care (ICU) patients with sepsis, although there is no convincing evidence for a survival benefit with hydrocortisone alone (23). The combination of hydrocortisone and fludrocortisone was found to reduce 90-day mortality (24).

### Sepsis, fluids and the microcirculation

It is important to appreciate the importance of alterations within the microcirculation (i.e., the arterioles capillaries and venules within tissue beds) in the pathogenesis of sepsis and septic shock (10). In critical illness and shock, shedding of the endothelial glycocalyx and endothelial dysfunction results in propagation of inflammation and the coagulation cascade (25). This leads to heterogeneity of vessel density and distribution of blood perfusion in tissues which is associated with organ dysfunction and worse outcome (26, 27). These changes persist despite optimization of systemic hemodynamic variables and oxygenation, a loss of so-called “hemodynamic coherence” (28–30). There is increasing interest in understanding the effects of resuscitation fluids on the microcirculation, and the relation to clinical outcomes (31).

### Clinical synthesis

Bedside assessment of the patient with sepsis requires attention to hemodynamic variables such as heart rate and blood pressure but also an appreciation of microcirculatory, cellular and metabolic factors on which tissue perfusion and organ function depend. Factors such as mental status, respiratory rate, skin perfusion and urine output are clinical features. The measurement of lactate is strongly recommended in patients with suspected septic shock (32) and forms part of the criteria for diagnosis (8), notwithstanding its limitations as a marker of hypoperfusion as previously discussed.

The rationale for administering an IV fluid bolus is to reverse potential hypovolemia and optimize venous return, with the aim of maximizing stroke volume and cardiac output. The assessment of volume status at the bedside in the emergency department is challenging. Non-invasive dynamic techniques such as echocardiography, pulse-pressure variation or bioimpedance cardiac output monitoring in combination with passive leg raising (PLR) or administration of a fluid bolus are increasingly employed (33). Static measures such as central venous pressure measurement are not reliable in determining fluid-responsiveness. Alternative approaches which have been studied include the assessment of skin capillary refill time (CRT) as a trigger for fluid resuscitation (34). Skin mottling is associated with poor outcome in sepsis (35, 36). Interestingly, one study found improvements in CRT following a PLR maneuver which occurred despite no changes in systemic circulatory variables (37). In a multicentre clinical trial of patients with septic shock, a strategy of CRT-guided fluid resuscitation was associated with less organ failure at 72 h compared to one guided by lactate, although no significant mortality benefit was observed (38). While the optimal approach to determining fluid responsiveness is debated, the relationship between bedside assessment and improved outcomes is unclear (39). In one study, only around 70% of ICU patients receiving a fluid bolus were deemed to be “fluid responsive” (40). Further, any hemodynamic improvements with fluid boluses are typically not sustained (41). This is supported by the finding of no difference in vasopressor requirement between ICU patients with septic shock randomized to a fluid restricted vs. standard fluid management strategy in a pilot feasibility trial (42).

## Early goal-directed therapy

The role of fluids and vasopressors came to prominence following the publication of a landmark, small, single-center, randomized trial of a protocolized resuscitation strategy termed early goal-directed therapy (43). The trial reported a 16% absolute risk reduction in mortality associated with the use of a resuscitation protocol that emphasized the administration of large volumes of fluid targeted at achieving a specified central venous pressure goal, followed by the use of vasoactive medication to achieve blood pressure and a central venous oxygen saturation (ScvO_2_) target. This resuscitation algorithm was subsequently adopted into international guidelines for the management of patients with septic shock (44). The results of this trial have not been replicated in multi-center studies. Three large trials conducted in the United States (45), Australia and New Zealand (46) and the United Kingdom (47), and an individual patient data meta-analysis that included a total of 3,723 trial participants in 138 centers did not confirm a reduction in morality with the use of early goal-directed therapy compared to usual care (48). There are a number of possible reasons for this. For example, the initial ScvO_2_ was higher in the validation trials, however, direct comparison is problematic since this was measured at baseline in Rivers trial whereas the initial measurement in the validation studies occurred post-randomization, when initial fluid resuscitation and antimicrobials had already been delivered. What is demonstrated is that IV fluid resuscitation had been adopted into standard practice, and that mortality rates in all arms of the validation studies were lower than in the intervention arm of the Rivers trial (Table 1).

**TABLE 1:** Mean intravenous fluid volumes and administered and in-hospital mortality in sepsis early goal directed therapy trials.

	Rivers (2001)		PROCESS (2014)			(ARISE 2014)		PROMISE (2015)	
	EGDT	Usual care	EGDT	PBST	Usual care	EGDT	Usual care	EGDT	Usual care
Fluids ED arrival to randomization (mL)	-	-	2,254	2,226	2,083	2,515	2,591	1,950	2,000
Fluids randomization to 6 h (mL)	-	-	2,805	3,285	2,279	1,964	1,713	2,000	1,754
Total 0–6 h (mL)	**4,981**	**3,499**	**5,059**	**5,511**	**4,362**	**4,479**	**4,304**	**3,950**	**3,754**
Fluid 6–72 h (ml)	8,625	10,602	4,458	4,918	4,354	4,274	4,382	3,623	3,981
Total 0–72 h (mL)	**13,443**	**13,358**	**9,517**	**10,429**	**8,716**	**8,753**	**8,686**	**7,373**	**7,735**
Initial ScvO_2_ ± SD (%)	49 ± 11	49 ± 13	71 ± 13	-	-	73 ± 10	-	70 ± 12	-
Mortality (%)	30.5	46.5	21	10.2	18.9	18.9	18.8	25.6	24.6

Bold numbers are cumulative totals.

Importantly, there was significant variation in resuscitation practices across these three large trials. While the baseline characteristics of the included populations in the trials were similar with respect to age and markers of severity of illness such as APACHE II score and lactate levels (48), there were differences in fluid and vasopressor therapy. For example, approximately 21% of participants enrolled in the ARISE trial (46) were receiving vasopressors at baseline, compared to 3% in the PROMISE trial (47). Trial participants randomized to usual care in the PROCESS trial (45) received on average 500 mL more IV fluid compared to those randomized to usual care in the ARISE trial (46). This variation in processes of care highlights the possibility that therapeutic strategies with a different emphasis on fluid administration compared to vasopressors may be a target to improve mortality for patients with septic shock.

## Evidence of harm with fluids

Liberal IV fluids with positive fluid balance in sepsis is associated with increased organ failure, intensive care length of stay and mortality (49, 50). Two randomized trials comparing liberal vs. conservative intravenous fluid resuscitation in children (51) and adults (52) with sepsis in Africa showed increased mortality in the treatment arms receiving higher IV fluid volumes, with the excess mortality being due to persistent shock (53). Potential mechanisms of harm relate to edema, hemodilution, exacerbation of endothelial glycocalyx shedding, reflex vasodilatation, release of natriuretic peptides and the flushing of inflammatory mediators from previously closed capillary beds. In line with this, recent experimental data suggests that fluid resuscitation preceding the start of vasopressors is associated with higher lactate levels and a paradoxical increase in vasopressor requirements when compared with an immediate start of vasopressor therapy without previous fluid administration (54). It is important to acknowledge important differences in population (high prevalence of malaria, anemia, HIV infection) and resources (limited healthcare infrastructure, lack of access to critical care) which prevent translation to high income countries. Nevertheless, several observational studies also suggest that increased volume of resuscitation fluids and net positive fluid balance is associated with mortality in sepsis (55–57).

## Restricted intravenous fluids

Observational and pre-clinical data support the hypothesis that IV fluid restriction during the resuscitation phase for septic shock patients, and maintenance of organ perfusion with vasopressors, may improve outcomes *via* an altered host inflammatory response (54, 58–60). The reduction in the host inflammatory response may be mediated by several mechanisms, including; (1) increased cardiac output by vasoconstriction-mediated increased preload, moving fluid from unstressed to stressed circulation (61) and by improving myocardial contractility (62); (2) increased microcirculatory perfusion in septic shock (63–66), especially when the baseline microcirculatory blood flow is abnormal (67) and; (3) improved regional distribution of blood flow (54). It is important to consider that the optimal strategy for fluid restriction may differ between individuals, and rather than a fixed volume approach, this should be tailored to the individual requirements across the different phases of care including initial resuscitation, optimization, stabilization and ultimately fluid elimination (68).

## Intravenous fluid restriction and early vasopressors

The purported advantages of restrictive fluid practices, in combination with earlier vasopressors, include more rapid restoration of blood pressure and organ perfusion, reduced tissue edema and decreased endothelial injury and acute renal injury associated with rapid fluid bolus administration. As noted previously, initiation of noradrenaline improves cardiac output *via* increased preload from increasing the volume of the stressed circulation and increasing myocardial contractility, as well as its effect as a peripheral vasoconstrictor (61).

Laboratory models of sepsis have shown improved mortality associated with the early administration of norepinephrine in septic shock (54). In a propensity matched cohort of 186 ICU patients with septic shock, Ospina-Tascon et al. reported that early vasopressors (<1 h) vs. delayed initiation (>1 h) was associated with a separation in total IV fluids at 6 h (900 vs. 2,000 mL, *P* < 0.001) and net fluid balance at 24 h (3,905 vs. 5,400 mL, *P* < 0.001) (67). Decreased mortality was also reported with early initiation (hazard ratio 0.31, 95% confidence intervals 0.17–0.57, *P* < 0.001). Similarly, Bai et al. found increased mortality in patients with septic shock when norepinephrine was commenced more than 3 h after the onset of shock (odds ratio 2.16, 95%confidence intervals 1.23–3.81, *P* = 0.0007) (69). Conversely, in a large, observational study evaluating a state-wide sepsis protocol in patients presenting to the emergency department (ED), in-hospital mortality was not associated with time to completion of an initial fluid bolus (30 mL/kg) up to 12 h after presentation (50). It is important to note, however, that any conclusions drawn from observational and retrospective studies are limited by factors such as indicator bias and unknown/unmeasured confounders and high quality, large-scale randomized clinical trials are required.

Traditionally vasoactive drugs have required to be administered *via* a central venous line, generally in an intensive care setting. This poses a practical and logistical barrier to early initiation of vasopressors. The potential risks associated with peripheral administration need to be balanced against harms associated with delay to restoring adequate perfusion. Several observational studies point to the safety of peripheral administration of vasopressors, at least during the first few hours (70). In the ARISE EGDT trial, vasopressors were initiated *via* a peripheral cannula in 42% of cases which was associated with a shorter time to commencement (71).

## Emerging evidence

Several small, randomized, pilot studies evaluating a conservative fluid approach have demonstrated the feasibility and safety of a restrictive fluid resuscitation strategy combined with earlier vasopressors in septic shock. An association with improved clinical outcomes compared with usual care has also been reported (72–74). The REFRESH trial evaluated restricted fluids and early vasopressors vs. usual care for initial resuscitation in 99 patients with sepsis-induced hypotension within 8 Australian ED. Fluid separation at 6 h was achieved (30 vs. 43 mL/kg, *P* < 0.001). The restricted fluid strategy was also associated with a shorter time to vasopressor initiation [34 (interquartile range 15–88) min vs. 150 (interquartile range 63–224) min] and an increased proportion of patients receiving a vasopressor within the ED (72 vs. 47%, *P* = 0.011) (72). Similarly, a small, single-center randomized trial of restricted fluids for 72 h after ED presentation with septic shock demonstrated decreased IV fluid resuscitation volumes compared to usual care (73); albeit no mortality differences were observed in either the REFRESH or RIFTS trial. Of note, the double-blind, randomized CENSER trial reported decreased cardiovascular complications (new-onset arrhythmia and cardiogenic pulmonary edema) and a trend to decreased 28-day mortality with an early fixed dose of norepinephrine for 24 h (0.05μg/kg/min) vs. standard care after presentation to the ED with sepsis-induced hypotension (75).

A large, multicentre, randomized trial of fluid restriction vs. standard fluid therapy in 1,554 patients admitted to ICU with septic shock (within 12 h of onset) has recently been conducted (Conservative vs. liberal fluid therapy in septic shock; CLASSIC) (76). Fluid separation after 1 day was minus 813 mL (median) in the restrictive vs. standard care group and minus 1,627 mL after 5 days. Ninety-day mortality was not different between treatment groups (42.3 vs. 42.1%); in the pre-specified sub-group stratified according to pre-randomized fluid volume, no heterogenous treatment effect was observed between the group receiving ≥ 30 mL/kg and those patients receiving < 30 mL/kg.

Notably, patients in CLASSIC required receipt of a vasopressor or inotropic agent to meet study eligibility and the intervention was not confined to the first 6–24 h after presentation; rather, the resuscitation strategy was delivered for the duration of the ICU stay. In contrast, there are 2 large multicentre, randomized trials currently evaluating a restricted fluids strategy compared to usual care wherein the intervention period is limited to the early resuscitation period (24 h) in patients presenting to the ED with sepsis-induced hypotension; CLOVERS (United States NCT03434028) and ARISE-FLUIDS (Australia and New Zealand, NCT04569942). The planned sample size for CLOVERS was 2,320 patients with the primary outcome being hospital mortality censored at 90-days. Enrolment was recently closed for futility following the second interim analysis. The ARISE FLUIDS is actively recruiting with a planned sample size of 1,000 patients and a primary outcome of days alive out-of- hospital to 90 days. A third, large, randomized trial, EVIS (United Kingdom NCT05179499) in 3,286 patients with septic shock is evaluating whether an early peripheral vasopressor infusion targeted to a mean arterial pressure > 65 mm Hg for 48 h improves survival to 30-days compared to usual care. The results of all three trials is eagerly awaited and will undoubtedly inform the early hemodynamic resuscitation of patients presenting to the ED with septic shock, particularly the initial fluid volume and the timing of vasopressors.

## Summary

The Surviving Sepsis Campaign guidelines recommend at least 30 mL/kg of fluid within 3 h as first-line therapy for sepsis-induced hypoperfusion or septic shock with the subsequent introduction of vasopressors. The evidence underpinning these guidelines is low quality and the recommendations are, accordingly, graded as weak. A growing body of evidence suggests that initial hemodynamic resuscitation with a restrictive fluid approach, combined with earlier vasopressors, may improve clinical outcomes, including mortality. Several large-scale, multicentre trials are currently evaluating such a strategy. High quality evidence from these trials will inform both bedside clinicians and future international guidelines. Until then, the optimal fluid dose and the timing of vasopressors remains uncertain.

## Author contributions

All authors listed have made a substantial, direct, and intellectual contribution to the work, and approved it for publication.
